# Establishing the College of Pathologists of East, Central and Southern Africa – The Regional East Central and Southern Africa College of Pathology

**DOI:** 10.4102/ajlm.v9i1.979

**Published:** 2020-06-03

**Authors:** Shahin Sayed, Rudo Mutasa, Ephata Kaaya, Victor Mudenda, Erasmus Rajiv, Edda Vuhahula, Jamilla Rajab, Robert Lukande, Edwin Walong, Angela Mutuku, Kenneth Fleming

**Affiliations:** 1 Department of Pathology and Laboratory Medicine, Aga Khan University Hospital, Nairobi, Kenya; 2 Department of Pathology, University of Zimbabwe, Harare, Zimbabwe; 3 Department of Pathology, Muhimbili University of Health and Allied Sciences, Dar es Salaam, United Republic of Tanzania; 4 Department of Pathology, University Teaching Hospital, Lusaka, Zambia; 5 Department of Chemical Pathology, Stellenbosch University, Stellenbosch, South Africa; 6 Department of Human Pathology, University of Nairobi, Kenyatta National Hospital, Nairobi, Kenya; 7 Department of Pathology, Makerere University, Kampala, Uganda; 8 College of Pathologists of East Central and Southern Africa, ECSA Health Community, Arusha, United Republic of Tanzania; 9 Green Templeton College, University of Oxford, Oxford, United Kingdom

**Keywords:** COPECSA, ECSA, pathology, sub-Saharan Africa, college

## Abstract

**Issues:**

The scarcity of pathologists in sub-Saharan Africa is a well established fact that is attributable to few training programmes in the region; this is further compounded by the lack of harmonised curricula, training and exams within and without member countries.

**Description of the intervention:**

Through the Association of Pathologists of East, Central and Southern Africa, the College of Pathologists of East, Central and Southern Africa (COPECSA) was formed with the clear-cut goal of establishing a regional and internationally recognised college to support and inform good quality medical and laboratory practice by promoting leadership, mentorship and excellence in the safe practice of pathology through training, exams, accreditation, advocacy and professional development for health.

**Lessons learnt:**

Since its inception in 2010, COPECSA has conferred fellowships to 120 practising pathologists in the East, Central and Southern Africa in partnership with international organisations; the college has been awarded five competitive grants and conducted several quality improvement workshops.

**Recommendations:**

This paper describes the journey that COPECSA has made towards standardising the practice and training of pathology in the East Central and Southern Africa region.

## Issues

Pathologists are medical doctors specialising in the study of the cause of disease and how it affects the body. They study these by examining changes in the cells, tissues, blood and other body fluids. Pathology is, therefore, a wide area of medical practice that encompasses several specialties including, but not restricted to: anatomical and surgical pathology, cytopathology, microbiology, haematology, chemical pathology, forensic pathology, immunopathology, molecular pathology, and genetic pathology. The list keeps expanding with advances in medicine. Pathology is crucial to the practice of medicine and therefore touches every facet thereof. Pathologists guide medical doctors on the right path for treating disease(s) and significantly contribute to research that advances medicine and devises new treatment. In summary, pathologists investigate the potential, presence, cause, severity, and progress of the disease. Additionally, they also contribute to monitoring the effects of treatment on diseases.^1^

It has been well established for some time now that there is a scarcity of pathologists in sub-Saharan Africa.^2^ This scarcity is compounded by a shortage of training programmes, with only about 80 cellular pathologists being trained each year.^3^ In addition, the variable standards of training in pathology have further increased the gap in the delivery of quality pathology and laboratory services. As an example, some training programmes in anatomical pathology suffer from a lack of student exposure to sufficient numbers of cases and case mix^4^ due to a paucity of standardised guidelines for competency training. This situation is further exacerbated by the unrealistic expectations placed on newly-qualified pathologists who are posted to large public hospitals without appropriate mentorship and supervision and are thus ill-prepared to manage complex laboratory functions. The founding of the regional college of pathology for East Central and Southern Africa (ECSA) was hence conceptualised in an effort to address both issues.

## Description of the intervention

The idea of a regional college of pathology in the ECSA region is probably as old as the Association of Pathologists of East, Central and Southern Africa (APECSA), which was established in 1990.^5^ Subsequently, in the United Nations Development Programme’s review of member countries’ progress towards the attainment of the Millennium Development Goals at the half-way stage (2007), it was acknowledged that, although the ECSA nations had made varied progress on Millennium Development Goals pertaining to the health of citizens, member countries were behind on the realisation of targets and that much more in terms of action, allocation of resources and sustained commitment was needed.^6^

In response to this, with respect to the training of pathologists, the members of APECSA decided to move towards the establishment of the College of Pathologists of East, Central and Southern Africa (COPECSA). A Steering Committee was constituted in 2008 to spearhead the establishment of the college and work on the constitution. The Steering Committee comprised members from seven of the nine ECSA countries. The proposed name of the college was adopted as the ‘College of Pathologists of East Central and Southern Africa’ with the acronym ‘COPECSA’.

The Steering Committee organised a consultative meeting on the establishment of COPECSA on 4 August, 2009, at Fairview Hotel, Nairobi, through sponsorship from the British Division of the International Academy of Pathology (BDIAP) and the United Kingdom’s Royal College of Pathologists (RCPath).

Members of the Steering Committee, APECSA Executive Officers and representatives from APECSA countries attended the meeting. In addition to expertise from overseas; expertise was sought from two other existing African colleges: the College of Pathologists of South Africa and the West Africa College of Pathologists. The host organisation, East, Central and Southern African – Health Community, was also represented. The meeting laid the foundation for the formation of COPECSA by deliberating on the Constitution, the structures of the future college, membership, registration fees, timelines for operationalisation and linkages with other colleges and institutions. It was agreed that COPECSA would be an autonomous body and the Steering Committee was proposed to act as an Interim COPECSA Council.

The Steering Committee presented a final draft Constitution and organisational structure for the college, which was adopted by the APECSA 2010 Annual General Meeting in Kampala, Uganda, where a resolution was passed that paved the way for the establishment of the college. The Constitution provided for pathologists in APECSA to be Founder Fellows.

**FIGURE 1:**
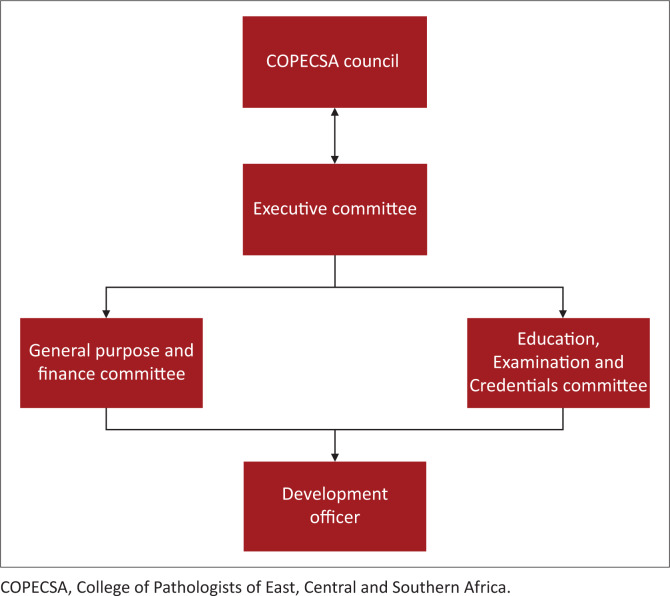
The College of Pathologists of East, Central and Southern Africa organogram.

The college was formally inaugurated at the same APECSA meeting in Kampala in September 2010; an executive council and members of the COPECSA Council were elected. A six-member Executive Committee, who form part of the 21-member Council drawn from the ECSA region, governs the college. The Executive Committee oversees the functions of the Education, Examination and Credentials Committee and the General Purpose and Finance Committee (Figure 1). A part-time development officer, a position that has been funded by the RCPath and BDIAP, runs the day-to-day administrative functions for the college.

## College of Pathologists of East, Central and Southern Africa – Structure and aims

The college is a professional body that draws its membership from pathologists registered and practising in the 16 member countries, namely: Kenya, Tanzania, Uganda, Rwanda, Burundi, Zambia, Zimbabwe, Ethiopia, Eritrea, South Africa, Malawi, Seychelles, Madagascar, Botswana, Zanzibar and Mauritius (Table 1). To foster collaboration among the pathology profession on the African continent, an executive decision was made to invite eligible pathologists from the West African College of Pathologists to become fellows of the College. Furthermore, in recognition of their contribution to the establishment of the college, honorary fellowships were bestowed upon individual fellows of the RCPath and the BDIAP. The college was established ‘to develop leadership and promote regional excellence in the practice of pathology and to be responsible for maintaining standards through training, examinations and professional development’.^5^ With an overarching aim for supporting the practice of pathology in the region, COPECSA strives to be recognised as a college that provides structure and leadership in the fields of both anatomical and clinical pathology in sub-Saharan Africa.

**TABLE 1:** Number of College of Pathologists of East, Central and Southern Africa fellows distributed across countries.

Country	Number of fellows	Number of pathologists in country^7^	Percentage of fellows over total number of pathologists in-country
Burundi	1	3	33
Botswana	1	6	17
Ethiopia	1	55	2
Ghana	1	30	3
Kenya	34	60	57
Malawi	1	9	11
Mozambique	1	4	25
Nigeria	28	170	16
Rwanda	2	5	40
South Africa	5	242	2
Tanzania	16	22	72
Uganda	13	24	54
United Kingdom	3†	-	-
Zambia	2	6	33
Zanzibar	1†	-	-
Zimbabwe	10	10	100

† , Honorary fellows.

Through ‘Excellence in Pathology for Better Health’ COPECSA seeks to contribute to the quality of health care services for citizens in each of the member countries and beyond, especially by means of the health-related Millenium Development Goals and, now, Sustainable Development Goals.

The Goal of COPECSA is to establish a regional and internationally recognised college that supports and informs medical and laboratory practice and ensures quality pathology in Africa. To make this vital contribution to healthcare and the provision of quality health services, COPECSA is committed through its Vision: ‘To develop and promote leadership and excellence in the safe practice of pathology through training, accreditation, advocacy and professional development for quality health’.^8^ Further, COPECSA as a college endeavours in practice to realise its critical mandate as stated in its Mission:

To develop a highly competent and ethical specialist workforce in Pathology able to provide quality laboratory services for diagnosis, prevention, and management of disease and also for research to international standards. [https://www.copecsa.org/about-us/].^8^

From the outset, the college recognised the importance of setting structures in place to operationalise functions. This process involved the development of the college constitution, a strategic plan, a robust competency-based curriculum, financial considerations and providing opportunities for research and training through leveraging its partnerships with various international organisations. The following sections highlight the various activities the college has undertaken, lessons learned along the way and achievements to date.

## College constitution

The Constitution highlights COPECSA’s Articles of Association where, ‘Pathology’ has been construed in the fullest and most inclusive sense, outlining in detail the college governance.^6^

## Strategic plan

Anchored on the college’s Vision and Mission statements, the 5-year Strategic Plan (2012/3–2017/8) charted the path for the future growth of COPECSA. This plan was focused on priorities that align goals towards upholding the core value of maintaining international standards in the safe practice of pathology within the region. The college identified its main strengths and weaknesses, while recognising opportunities for growth (Box 1). During the preparation of the strategic plan, arious approaches were deliberated upon in order to address the core issues identified and to mitigate the threats it faces in the current environment (Table 2).

Box 1 College of Pathologists of East, Central and Southern Africa strengths, opportunities, weaknesses and threats analysis, 2012.

**Table:** 

Strengths	With current overseas members, as well as partnerships and affiliations, the college has been able to provide short continuous improvement courses, specifically in cancer diagnosis.
Weaknesses	Limited sources of revenue as currently the membership dues are the most critical source of income, making up over 90% of the organisation’s revenue. Lack of full-time dedicated staff to facilitate the daily running of the organisation. Not all countries within the ECSA region have adopted or recognised COPECSA’s curricula and standards. Adoption is essential if harmonisation of training standards is to be achieved across the region. Distances between executive members makes physical meetings difficult. Lack of a uniform currency and stringent exchange control regulations in member countries makes remittance of college subscriptions a challenge.
Opportunities	Through its focus on provision of educational, training and continuous professional development, COPECSA can partner with internationally-accredited institutions to raise its profile as the standard-setting body within the ECSA region. With the increase in cancer incidence in the region, the role of pathology has taken centre stage. The college, in its advisory capacity, can support governments in development and implementation of standards of practice in pathology. The college, through its various partnerships, can engage in research to inform policy for pathology practice in the region. The college has resolved to work with other ECSA-HC constituent colleges to ensure that there is acceptance across the board.
Threat	Competition from emerging pathology and laboratory medicine associations in the ECSA region and abroad.

COPECSA, College of Pathologists of East, Central and Southern Africa; ECSA, East Central and Southern Africa; ECSA-HC, East Central and Southern Africa Health Community.

**TABLE 2:** College of Pathologists of East, Central and Southern Africa Strategic Plan (2012–2018).

Issue	Strategy	Strategic objectives	Achievements and planned activities
1. Lack of an established and operational COPECSA secretariat	Establish and operationalise COPECSA as a vibrant college.	**SO 1(a):** Set up a physical location and recruit secretariat staff for COPESCA by the end of June 2012.	Officially launched strategic plan to include: conferment of fellowship to founder members, AGM to commission the secretariat. Completed registration of the college. Completed negotiation and identified physical office with ECSA-HC.
		**SO 1(b):** Develop and implement organisation policies, procedures and systems for COPECSA by the end of 2012 (human resources, finance, administrative policies and procedures).	Developed the job descriptions for secretariat staff. Advertised and recruited staff. Opened a secretariat bank account. Drafted policies and procedures manual of operation and had them approved by the Council. Branded, marketed and created visibility. Developed and published a College Bulletin/Journal.
2. Need for quality Pathology Fellowship programmes in COPECSA member countries	Harmonise pathology training in member countries.	**SO 2(a):** Develop and implement quality Pathology Fellowship programmes in the member countries by the end of 2012.	Reviewed existing pathology training programmes from COPECSA member countries. Developed the COPECSA Fellowship Curriculum. Established COPECSA members existing competency domains. Established criteria for college examination. Developed criteria for training and examination centres. Established criteria for trainers and examiners. Conduct training of examiners. Established calendar of the college.
		**SO 2( b):** Accreditation of COPECSA as a CPD provider by the member country medical practitioner and dentist boards or councils by end of 2013.	Developed COPECSA registry (point rating etc.). Lobby medical boards and councils for accreditation of COPECSA as a CPD provider.
3. Low level of engagement of Pathology in national policy & advocacy issues.	Position COPECSA in key policy and decision-making bodies to inform and influence policy.	**SO 3:** Identify, inform advocate and engage for the formulation and improvement of at least one laboratory policy in each member country over the planning period.	COPECSA members to sit on policy bodies in each member country. This has been achieved in some countries, such as Kenya, Tanzania and Zimbabwe. Provide technical know-how in policy making. Document and archive reports and information.
4. Inadequate resources	Develop a fundraising strategy.	**SO 4:** Develop and implement a fundraising and resource mobilization strategy within the first Strategic Plan year (2012).	**Activities:** Developed the fundraising strategy. Recruit 30% eligible persons each year into COPECSA. Write and submit at least one grant proposal per year. Fundraise for the strategic plan and COPECSA activities.
5. Lack of formal partnerships	Develop a partnership strategy.	**SO 5:** Develop and launch the implementation of a COPECSA partnership strategy by the third quarter of 2012.	**Activities:** Identified different institutions and organisations to partner with. Developed a MoU/partnership agreement template. Formalise the existing partnership with RCPath. Identify areas of collaboration and partnership with like-minded organisations.
			**Proposed Partners:** Association of Pathologists of East, Central and Southern Africa, East Central and Southern College of Health Sciences, African Society for Laboratory Medicine, RCPath, and College of Medicine of South Africa, West African College of Postgraduate Studies, Med Tech Boards, International Academy of Pathology, Universities and others.

AGM, Annual General Meeting; COPESCA, College of Pathologists of East, Central and Southern Africa; CPD, Continuous Professional Development; MoU, Memorandum of Understanding; RCPath, Royal College of Pathologists; SO, Strategic Objective.

## Curriculum

The harmonised curriculum sets out the minimum standards and competencies for training and residency programmes in anatomical, clinical and general pathology. The curriculum seeks to harmonise the knowledge, skills and behaviours that respective trainees should acquire during their training and residency in the region.^8^

The required qualification to become a fellow is an undergraduate medical degree (Bachelor of Medicine and Chiropody [MBChB], Bachelor of Medicine and Surgery [MBBS], or equivalent) qualification. After training in pathology at accredited training institutions, the trainee is certified and conferred a fellowship upon successfully passing the COPECSA exams.

In order to kickstart the college, founding fellows had to be inaugurated by nomination in order to have a cohort of well-known and recognised academics in the region who could lay the foundation. Hence, the inaugural fellows were capped in Arusha, Tanzania, by senior officials of international sister bodies that had helped set up the college.

However, it was soon realised that the original cohort was not broad enough to represent: (1) all the regions of sub-Saharan Africa and (2) all the specialties in pathology that would allow the college to mount examinations in all. For this reason, a second cohort of fellows was capped in Kigali, Rwanda, in 2016.

Going forward, however, the thrust of the college and award of fellowship will largely be by examination. It has taken the college three years to initiate examinations in order to ensure that the standard of the exam is set at the right level from the very beginning. All the necessary stakeholders have been consulted and due diligence performed. The college is now ready to mount its first examination before the end of 2020.

In an accompanying article^9^, we articulate the need for a harmonised curriculum that can serve the region, as has been demonstrated by the lessons learned from the Zambia MMed programme in cellular pathology. It is notable that COPECSA’s Anatomical Pathology Curriculum has recently been adopted in full by the Ministry of Health and Ministry of Education in Zambia. In Tanzania, COPECSA’s curricula have been presented to the Tanganyika Medical Council for consideration and in Kenya, the Ministry of Health and the Kenya Medical Practitioners and Dentists Board are working with stakeholders to draft a policy paper for the adoption of a collegiate system for all medical specialities. However, much remains to be done to ensure adoption and recognition throughout the ECSA region.

## Sources of funding

The College of Pathologists of East, Central and Southern Africa is currently dependent on its membership subscriptions. However, the college has been supported in its various activities by both the RCPath and BDIAP. Both BDIAP and RCPath have also supported the position of a part-time development officer for the college. This was with the understanding that the college must become self-sustaining/sufficient in the near future.

## Achievements to date

### Conferment

**FIGURE 2:**
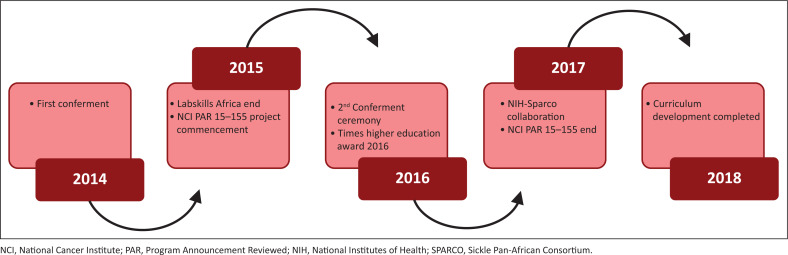
College of Pathologists of East, Central and Southern Africa achievements.

The college held its first conferment ceremony in 2014 in Arusha, Tanzania, where 64 pathologists across the ECSA region had their degrees conferred (Figure 2). Subsequently in 2016, the college conferred fellowships on 56 pathologists in Kigali, Rwanda.

### Development of a curriculum for the region

The Education Examination and Credentials Committee of the college has worked towards developing curricula in general pathology, anatomical pathology and clinical pathology. The curriculum development process included convening a Curriculum Development Committee to identify critical issues, trends in the content area and assess regional needs. Once the programme and course goals and objectives were set, deliberations were held to identify resource materials for programme implementation. Assessment tools and instruments were developed to measure student progress. The college is currently in the process of implementing its curriculum.

### Partnerships and projects

In partnership with various international organisations (Box 2), COPECSA has successfully implemented programmes and projects in which more than 100 pathologists have been trained in various aspects of pathology and laboratory medicine service.

Box 2 Current active College of Pathologists of East, Central and Southern Africa partnerships.

**Table:** 

The Royal College of Pathologists British Division, International Academy of Pathology	Provided support for establishment of COPECSA. Supported COPECSA curriculum development. Provided support for part-time development officer. Supports faculty to offer short master courses alongside APECSA conferences.
American Society for Clinical Pathology	Subsidised courses for Fellows. Specifically, the Lab Management course offered by the ASCP where each successful participant from COPECSA enjoys a 10% discount for the course.
African Strategies for Advancing Pathology	Collaboration to provide training to pathologists on improving cancer diagnosis and cancer staging in accordance with international guidelines.
East, Central and Southern Africa College of Health Sciences	The ECSA College of Health Sciences is an umbrella body which houses and regulates all professional colleges in the ECSA region, including COPECSA guided curriculum development by establishing minimum standards and guidelines.
East Central and Southern Africa	Supports the College secretariat at Arusha, Tanzania.

APECSA, Association of Pathologists of East, Central and Southern Africa; ASCP, American Society for Clinical Pathology; COPECSA, College of Pathologists of East, Central and Southern Africa; ECSA, East Central and Southern Africa.

### Programmes and projects

**The LabSkills Africa programme ($1 100 000.00):** This programme was designed to improve the standards and quality of both pathology and laboratory medicine services. The college partnered with RCPath, BDIAP, Aga Khan University Hospital Nairobi, Stellenbosch University, ECSA Health Community and five in-country associations (https://www.rcpath.org/international/projects/labskills-africa.html). This programme was a 30-month health systems strengthening initiative funded by the United Kingdom’s Department for International Development, whereby 20 laboratories in Kenya, Uganda, Tanzania, Zambia and Zimbabwe participated in the use of integrated skills training, knowledge transfer, leadership development and mentoring for the following key tests: rapid HIV, rapid malaria, peripheral blood film, haemoglobin/haematocrit estimation, urinalysis and tuberculosis. At the culmination of the LabSkills Africa programme, a combined population of 110 million people was served, and 1.7 million tests performed in the laboratories (https://www.rcpath.org/international/projects/labskills-africa.html). Also, the programme was able to train and mentor 100 pathologists, biomedical scientists, laboratory technologists and technicians across the 20 laboratories. The COPECSA/RCPath partnership was feted as a recipient of the TIMES Higher Education Award in the category International Collaboration of the Year (https://www.timeshighereducation.com/news/times-higher-education-awards-2016).^10^

**National Cancer Institute Program Announcement Reviewed by an Institution 15–155 ($56 000.00):** In 2016–2017, COPECSA, in partnership with the University of Colorado Cancer Centre and African Strategies for Advancing Pathology, was the recipient of a National Cancer Institute Program Announcement Reviewed by an Institution (15–155) initiative, a regional programme for improving anatomical pathology to support cancer care. This was a 17-month research strategy that focused on evaluating the best approaches to training pathologists and senior residents in ECSA with high quality standardised cancer diagnosis in order to determine which approach is most effective at improving the expertise of the pathology workforce in low- and middle-income countries, and share the lessons learned to contribute to future training efforts.^6^ This programme engaged 17 pathology departments and trained 52 pathologists and senior residents in institutions from: Zimbabwe, Kenya, Uganda, Tanzania, Zambia, Rwanda, Burundi, Malawi, Madagascar, Mozambique, and Botswana. A follow-up training workshop was held on 17–19 November, 2018 in Nairobi, Kenya, funding for which was awarded under the National Institutes of Health R13 grant mechanism.^6^ An additional 20 practising pathologists and senior pathology residents from eight countries were trained in staging of breast, cervical, ovarian, colorectal, stomach, endometrial and head and neck cancers. The programme was unique in that international faculty members were paired with local faculty members in the planning and designing of the course curriculum in order to build the capacity of the local faculty to deliver the course content in future workshops.^11,12^

**Cytopathology short course:** The college, in partnership with the International Academy of Cytology, trained a total of 27 practising pathologists from the region in image-guided fine-needle aspiration biopsy in Nairobi, Kenya. Faculty members from the University of California, San Francisco (United States) and Notre Dame University, Sydney (Australia) led the course.

**National Institutes of Health – Sickle Pan-African Research Consortium Collaboration ($634 807.00):** COPECSA supported the application for a National Institutes of Health grant by one of its fellows. The Sickle Pan-African Research Consortium brings together clinicians, academicians and scientists with existing sickle cell disease programmes, from the hub in East Africa (Tanzania) and collaborative consortium sites in West Africa (Ghana, Nigeria), which will expand to form the Sickle Pan-African Network by involving 22 sites in 17 countries. The main goal is to reduce the public health burden (mortality and morbidity) of sickle cell disease in Africa while at the same time establishing the capacity for further research. The aim is for this research is to contribute to scientific knowledge in order to find a cure for sickle cell disease by striving to reach an understanding on how a monogenic disease can have such heterogeneity in the region.

It has taken COPECSA more than 5 years to get off the ground. Many lessons have been learnt along the way as the college begins to establish its footprint. Currently, COPECSA covers 13 countries in sub-Saharan Africa, which may have been an overly ambitious plan. Political instability in some member states and communication challenges across borders have been problematic. The engagement of stakeholders at multiple levels in member countries has been a slow and onerous process due to limited funding sources and the lack of a dedicated full-time secretariat. The dependence of the college on international partner support for running the affairs of COPECSA has been fraught with challenges as the college, over the years, has become overly reliant on external funding to support its day-to-day functions. Furthermore, there have been varying levels of success in persuading university authorities in some member countries of the benefits of adopting a harmonised curriculum and examination process. Uncertainties regarding the recognition and registration of fellows by individual country medical boards has further impeded progress.

Currently, the unpredictable financial sustainability of COPECSA and a lack of adequate visibility and assurance of employment opportunities have been barriers to attracting fellows to the college. In order to mitigate some of these challenges, in some member countries, like Kenya and Tanzania, the college is working with other ECSA colleges to ratify their curricula and examinations with the countries’ medical boards. For a long time, the Executive Committee were under the misperception that college curricula were regulated under the Commission of University Education rules and therefore needed to be approved by the Commission of University Education before roll out. The Commission of University Education requirements include appropriate physical infrastructure, which was almost impossible to fulfil as the collegiate system was envisioned to be based on apprenticeship at existing accredited training facilities followed by a final common exit exam upon completion of college curricular requirements. A lot of time was thus wasted in the process of understanding the regulatory framework for each country. The recognition that pathology is a highly-specialised discipline meant that only select training facilities across the ECSA region would have the faculty, volumes, case mix and equipment for adequate exposure to specialised techniques such as immunohistochemistry, cytology, and medical and forensic autopsies. Prospective candidates would thus be expected to take responsibility for their own training costs at these accredited centres, while the college provides logistical support for visa and accommodation options. To avoid overburdening the current few training facilities with new students, a decision was made by the Executive Committee to restrict fellowship exams to recent MMed pathology graduates and practising pathologists.

Lessons learned

**Table:** 

The Executive Committee recognised the importance of drafting a list of benefits that would attract qualified pathologists to join the college. The benefits include:
Provision of opportunities to influence the development of standards of professional training and practice.
Access to educational resources to help stay current with advances in laboratory medicine.
Eligibility for discounts on registration fees for the college’s scientific meetings.
Eligibility to become an examiner for the college.
Government representation and advocacy on key issues of importance to the specialty.
Opportunity to get involved in the governance of the college.
Provision of guidance on consent and ethics with matters related to pathology specialties.
Provision of guidance and assistance with workforce planning, workload management and other issues pertinent to the profession.
Professional enrichment through volunteer opportunities and worldwide peer recognition – the Fellow of the College of Pathologists (FCPath) distinction.
Offer of reduced membership rates for trainees.
Provision of opportunities to take part in local, national and international meetings.
Access to development grants.
Incentive prizes to trainees.
Access and opportunity to participate in basic and senior management courses.
Exertion of national and international influence through representation on other organisations.
Provision of advice to national advisory panels on quality assurance.
Being a member on Joint Committees with other professional organisations.
Being involved in the development of Best Practice Guidelines and use of Diagnostic Tests.
Supporting the career development of its members.
Taking part in the development of Position Statements on behalf of COPECSA.

The Executive Committee also noted that the current lack of clarity on cross-border recognition of college credentials is one of the issues that needs to be addressed, in order to attract more members to COPECSA.

## Future plans

### Examinations

The first sitting of COPECSA’s Fellowship Examinations will take place in July 2020. Before July 2020, COPECSA proposed to undertake the following activities in preparation for the first sitting:

- Publish and promote the COPECSA curricula.
- Recruit and train COPECSA examiners.
- Launch a series of webinars to provide key stakeholders, institutions, trainees and prospective candidates with an introduction and overview to COPECSA’s curricula, training standards and examinations.
- Establish a network of recognised training institutions and examination centres throughout the region.
- Develop an accreditation process for training institutions, wishing to be accredited by COPECSA.
- Launch COPECSA’s Trainee Membership category.
- Applications to sit the 2020 COPECSA Fellowship Examinations would open at the end of February 2020.

### Recommendations

The establishment of a regional college of pathology in ECSA is a first step in harmonising pathology training and standardising the practice of pathology within the region. Through sharing of resources among member states, the college aims to maximise scarce human resources and infrastructure. Furthermore, the proposed accreditation of training sites lends itself to improvement in the quality of pathology training and subsequent practice.

The college strongly recommends partnerships with like-minded regional and international pathology associations as a strategy that is vital for the successful implementation of the college agenda.

A key recommendation of the college is measurement of improvement in health services that can be ascribed to the establishment of COPECSA. The value of a pathologist in clinical practice has already been highlighted. The college plans to institute monitoring and evaluation activities to measure its impact on improved health service provision and overall health outcomes within the ECSA region.
